# Incidence, severity, and predictors of citrate accumulation during continuous kidney replacement therapy in the critically ill

**DOI:** 10.1186/s13054-025-05691-2

**Published:** 2025-11-03

**Authors:** Mattia M. Müller, Alexa Weber, Jan Bartussek, Jasmin Bachmann, Alix Buhlmann, Gabor Kadler, Caroline John, Rolf Erlebach, Daniel A. Hofmaenner, Rea Andermatt, Marc Thorsten Schmidt, Reto A. Schuepbach, Christoph C. Ganter, Pedro D. Wendel-Garcia, Sascha David

**Affiliations:** 1 Institute of Intensive Care Medicine, University Hospital and University of Zurich, Zurich, Switzerland; 2 Institute of Intensive Care Medicine, University Hospital Zurich, Zurich, Switzerland; 3 Department for Quantitative Biomedicine, University of Zurich, Zurich, Switzerland; 4 Division of Cardiothoracic Anaesthesia and Intensive Care Medicine, Department of Anaesthesiology, General Intensive Care and Pain Medicine, Medical University Vienna and Vienna General Hospital, Vienna, Austria; 5 Department of Nephrology and Hypertension, Medical School Hannover, Hannover, Germany

**Keywords:** Citrate accumulation, CKRT, Kidney failure, Dialysis, ICU, Critical care, Lactate

## Abstract

**Background:**

Regional citrate anticoagulation (RCA) is the recommended anticoagulation strategy for continuous kidney replacement therapy (CKRT). However, the safety of RCA in patients with liver dysfunction and/or shock remains controversial due to the risk of citrate accumulation. This study assesses the associations of citrate accumulation with liver dysfunction, circulatory shock, and mortality, and investigates lactate and the vasoactive inotropic score (VIS) as early predictors.

**Methods:**

This retrospective cohort study included critically ill patients requiring RCA-based CKRT between January 2018 and March 2022. Lactate, VIS and parameters of organ failure were investigated as predictors of citrate accumulation. An albumin-corrected total calcium to ionized calcium ratio ≥ 2.5 was used to define citrate accumulation. Regression models were employed to investigate the association of predictors with outcomes.

**Results:**

Nine hundred eleven patients were included, citrate accumulation was observed in 159 individuals (17%). Factors related to liver dysfunction, but not circulatory shock, were attributed to citrate accumulation. After multivariable adjustment, citrate accumulation was not associated with mortality. Lactate measured before onset of CKRT showed an improved discriminative performance compared to the VIS. The odds of citrate accumulation increased by 2.34 (CI 1.94–2.85, *p* < 0.001) for each one-unit increase in lactate on the logarithmic scale (log mmol/L). The probability for citrate accumulation ranged from 3.3 (CI 2.06–5.28) % at lactate levels of 0.3 mmol/L to 59.8 (CI 48.88–69.78) % at levels of 25 mmol/L.

**Conclusion:**

Lactate is a reliable predictor for assessing the risk of citrate accumulation in patients undergoing CKRT. Further research is needed to develop and validate predictive algorithms for various anticoagulation strategies to offer reliable support for personalized decision-making in clinical practice.

**Supplementary Information:**

The online version contains supplementary material available at 10.1186/s13054-025-05691-2.

## Introduction

Regional citrate anticoagulation (RCA) during continuous kidney replacement therapy (CKRT) has been shown to be superior in reducing bleeding complications and extending filter lifespan compared to systemic heparinization or the complete avoidance of anticoagulation [1–4]. Consequently, the *Kidney Disease: Improving Global Outcomes* (KDIGO) guidelines recommend RCA over other modalities in the absence of contraindications [5]. However, in patients with circulatory shock or impaired liver function, RCA might lead to citrate accumulation and subsequent toxicity [6]. The underlying pathophysiological mechanism of citrate accumulation is commonly thought to be mitochondrial dysfunction, resulting in impaired citrate metabolism [7]. To date, the impact of citrate accumulation on patient outcomes remains poorly understood. Moreover, there are no specific recommendations for reliable predictors to assess an individual’s risk of citrate accumulation. Parameters of impaired systemic circulation and/or severe liver dysfunction, such as lactate levels and vasopressor requirements, have been suggested as predictors, but no validated benchmark levels currently exist to guide clinical decision-making [8–10].

This study aimed to investigate the incidence of citrate accumulation in critically ill patients undergoing RCA-based CKRT, evaluate its association with liver injury, circulatory shock and patient outcomes, and assess pre-CKRT plasma lactate levels and the Vasoactive Inotropic Score (VIS) as early predictors.

## Materials and methods

### Ethical approval

The study was conducted in accordance with established ethical standards and the Declaration of Helsinki. The Cantonal Ethics Commission of Zurich, Switzerland (Kantonale Ethikkommission Zürich, BASEC-ID: 2022 − 01938) reviewed and approved the study protocol. Due to the retrospective nature of the study and in compliance with local regulations, informed consent was waived. Patients were excluded if they had explicitly declined the use of their medical data either verbally or in writing.

### Patient population

Patients hospitalized between January 2018 and March 2022 at the Department of Intensive Care Medicine at the University Hospital in Zurich were screened for eligibility. Inclusion criteria were a documented CKRT in the electronic patient records (EPR), use of RCA at CKRT start and age ≥ 18 years. Patients were excluded if the type of anticoagulation was not identifiable within EPRs, if an anticoagulation other than RCA was used upon initiation of CKRT or if patient deceased before start of the follow-up period. Protocols for the initiation and adjustment of CKRT devices are provided in the Supplemental Table S1a–d.

### Predictors

Arterial lactate levels and the VIS were considered as principal predictors. Patients with a VIS > 0 were defined as in circulatory shock for the purpose of this study. Bilirubin, factor V (FV), and the international normalized ratio (INR) were used as parameters of impaired liver function. These factors were selected based on the literature as they are the most widely recognised indicators of liver injury in commonly used scores (e.g. bilirubin in the SOFA score) and reliable markers of impaired hepatic synthesis (INR, FV) [11–13]. Other liver parameters (e.g. ASAT, ALAT) were not included to avoid collinearity in the prediction models. Central venous oxygen saturation (ScvO₂) and the arteriovenous CO₂ gap (ΔavCO₂) were included as surrogate markers of global tissue perfusion, as low ScvO₂ and elevated ΔavCO₂ are well-established indicators of impaired tissue oxygenation and perfusion [14, 15]. The following covariables were a priori defined for adjustment based on a directed acyclic graph: age, sex, surgical intervention prior to admission, pO2/FiO2 (P/F)-ratio, platelet counts, creatinine, pH, leucocytes, C-reactive protein (CRP), blood-to-dialysate + substitute flow ratio (B-DS-ratio), Glasgow Coma Scale (GCS), and modus of CKRT (continuous venovenous hemodialysis (CVVHD) or continuous venovenous hemodiafiltration (CVVHDF)). The B-DS-ratio was calculated using the following equation: [blood flow/(dialysate flow + substitution flow)], where blood flow and dialysate flow represent the set flow rates in ml/min for blood and dialysate in CVVHD and CVVHDF devices, and substitution flow represents the filtration rate in ml/min in CVVHDF devices.

### Outcome parameter

A total albumin-corrected-to-ionized calcium ratio (T/iCa) ≥ 2.5, was used as a marker of citrate accumulation for the primary endpoint [16]. ICU mortality and length of ICU stay were considered as secondary endpoints. T/iCa and ICU mortality were prespecified endpoints, whereas ICU length of stay was added during the analysis. The follow-up period for the primary endpoint began one hour after the initiation of CKRT, allowing time for CKRT to reach a steady state, and continued for up to 14 days or until the earliest occurrence of the following events: discontinuation of RCA, discontinuation of CKRT due to renal recovery, switch to intermittent hemodialysis, or death. Secondary endpoints were assessed for the duration of ICU stay.

### Data collection

Patient characteristics at day of Intensive Care Unit (ICU) admission were extracted from EPRs. Lactate and vasopressor measurements taken one hour before the start of CKRT, were extracted as principal predictors. Vasopressors were transformed into the VIS score with the following equation: dopamine (µg/kg/min) + dobutamine (µg/kg/min) + 100 × epinephrine (µg/kg/min) + 100 × norepinephrine (µg/kg/min) + 10 × milrinone (µg/kg/min) + 10,000 × vasopressin (U/kg/min) [17]. Laboratory parameters measured closest to one hour before start of CKRT were used as covariables. T/iCa was calculated using the first documented measurements for each 8-hour interval, starting 1 h after the initiation of CKRT, using the following formula: Total albumin-corrected calcium/ionized calcium. First documented lactate levels and covariables, along with the average vasopressor infusion rate, were extracted for each 8-hour period to assess dynamic changes over time.

### Reporting

The reporting followed the Strengthening the Reporting of Observational Studies in Epidemiology (STROBE) checklist from the EQUATOR Network.

### Statistic

Baseline patient characteristics were collected at ICU admission and are reported as medians with interquartile ranges (IQR), absolute numbers, and percentages, as appropriate. Quantile-quantile plots were used to assess data distribution. Lactate values, VIS and covariables were log- or square root-transformed to approximate a normal distribution. A Kaplan-Meier curve was generated to visualize the cumulative incidence of citrate accumulation over time.

Binominal logistic regression models were employed to investigate the association between predictor variables and the primary endpoint, both in an unadjusted model and one adjusted for predefined covariables. Results are presented as odds ratios (OR) with 95% confidence intervals (CI). Areas under the receiver operating characteristic curve (AUC) and DeLong test were used to assess the performance of predictive models. Sensitivity, specificity, positive- and negative predictive values as well as Youden Index were calculated for different lactate thresholds. Linear mixed-effects models were established to evaluate the longitudinal trajectories of predictors in association with the primary endpoint. Binominal logistic regression models were used to assess the association of citrate accumulation with ICU mortality. Analysis was conducted in R version 4.2.2 (R Foundation for Statistical Computing, Vienna, Austria) using the following packages: survival, survminer, contsurvplot, lme4, lmerTest, merTools, ciTools, splines, pROC.

## Results

### Patients

Between January 2018 and March 2022, 1,858 patients hospitalized at the Institute of Intensive Care Medicine of the University Hospital of Zurich required kidney replacement therapy. After reviewing the cohort based on the exclusion criteria, 911 patients remained eligible for the final analysis. CKRT was initiated in 572 patients using the multiFiltrate device (Fresenius Medical Care, Bad Homburg, Germany), while the Prismaflex System (Baxter, Deerfield, USA) was used in 293 individuals. For 46 patients, the specific device was not documented. Table 1 presents an overview of patient characteristics. Inclusion process is delineated in Supplementary Fig. S2.

**Table 1:** Patient characteristics at ICU admission, CKRT settings and outcome parameters

Characteristic	Overall, *N* = 911	Citrate accumulation	
		No, *N* = 752	Yes, *N* = 159
Age, years, median (IQR)	63 (54–72)	63 (54–72)	64 (55–72)
Sex, n (%)			
F	260 (29)	194 (26)	66 (42)
M	651 (71)	558 (74)	93 (58)
Chronic kidney disease, n (%)	334 (37)	281 (38)	53 (34)
End stage renal disease, n (%)	92 (10)	80 (11)	12 (7.6)
Chronic lung disease, n (%)*	189 (21)	165 (22)	24 (15)
Acute respiratory failure, n (%)	323 (36)	273 (37)	50 (32)
Coronary heart disease, n (%)	322 (36)	280 (37)	42 (27)
Acute cardiovascular failure/shock, n (%)	405 (45)	327 (44)	78 (49)
Arterial hypertension, n (%)	416 (46)	360 (48)	56 (35)
Vascular disease, n (%)	313 (35)	261 (35)	52 (33)
Diabetes mellitus, n (%)	231 (26)	200 (27)	31 (20)
Chronic liver disease, n (%)	236 (26)	175 (23)	61 (39)
Acute liver injury, n (%)**	212 (24)	140 (19)	72 (46)
Trauma, n (%)	17 (1.9)	16 (2.2)	1 (0.6)
Neurological disease, n (%)	50 (5.5)	45 (6.0)	5 (3.2)
Surgical intervention before ICU admission, n (%)	412 (46)	345 (46)	67 (42)
SOFA at ICU admission, median (IQR)	12 (9–15)	12 (9–14)	13 (11–17)
SAPS, median (IQR)	55 (43–69)	54 (43–68)	58 (45–74)
CKRT modality at start, n (%)			
CVVHD	574 (67)	477 (67)	97 (64)
CVVHDF	284 (33)	230 (33)	54 (36)
Invasive mechanical ventilation, n (%)	697 (77)	584 (78)	113 (72)
ICU mortality, n (%)	348 (38)	256 (34)	92 (58)
Hospital mortality, n (%)	396 (44)	292 (39)	104 (66)

IQR = Interquartile ranges, CKRT = Continuous kidney replacement therapy, CVVHD = Continuous venovenous hemodialysis, CVVHDF = Continuous venovenous hemodiafiltration

*Chronic lung disease encompasses all chronic diseases of the lungs, including chronic obstructive pulmonary disease, chronic bronchial asthma, pulmonary hypertension, interstitial fibrosis, and other restrictive pulmonary disorders

**Acute liver disease encompasses acute hepatitis, acute liver failure, acute-on-chronic liver failure and acute decompensated liver cirrhosis

### Citrate accumulation

**Fig. 1:**
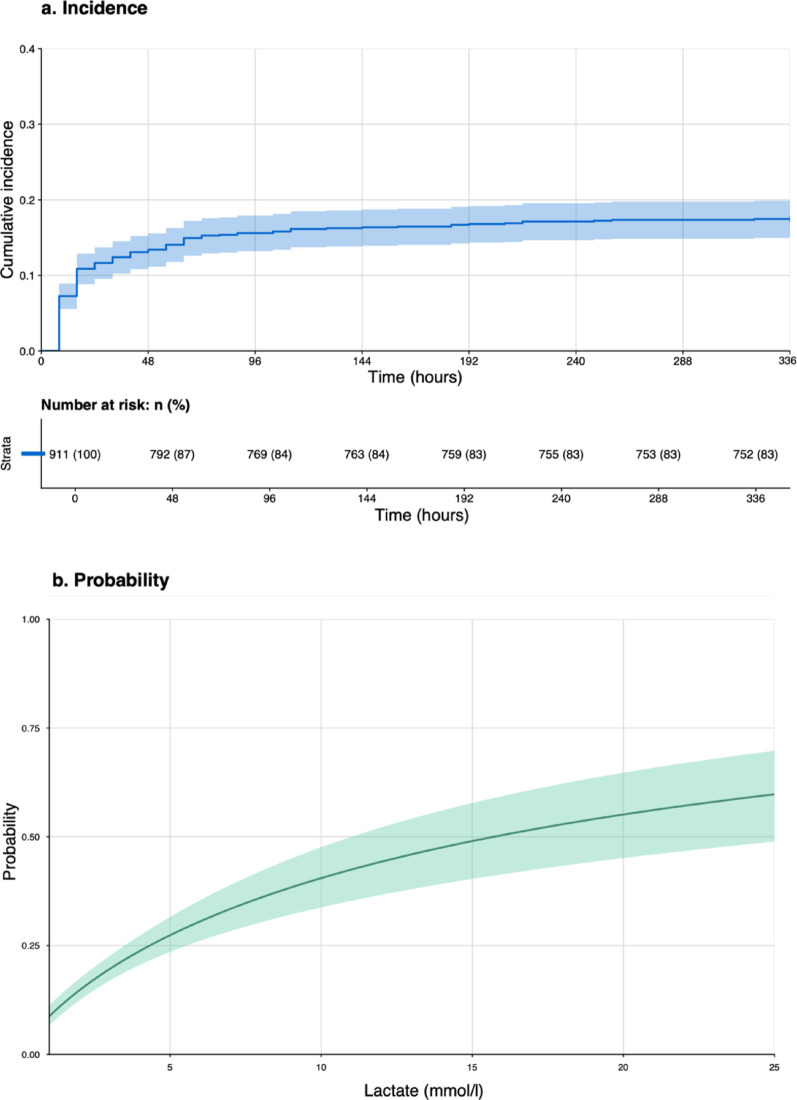
Incidence and probability of citrate accumulation based on lactate levels at start. (**a**) Shows how likely the patients are to experience citrate accumulation within 336 h (14 days) after start of CKRT (blue line = cumulative incidence, blue shaded = 95% confidence Interval). (**b**) Represents the probability of experiencing citrate accumulation within the first 14 days after the onset of CKRT, based on lactate levels measured prior to initiation. Lactate levels were transformed on a logarithmic scale for model development and then converted back to a continuous scale for improved interpretability (green line = predicted probability of citrate accumulation, green shaded = 95% confidence interval)

Of the 911 patients enrolled, 159 (17%) reached a T/iCa ≥ 2.5 indicative for a citrate accumulation during the 14-day follow-up period. The median time from the initiation of CKRT to the onset of citrate accumulation was 16 [8–44] h. Figure 1a. illustrates the cumulative incidence of events over time. The incidence of competing events and the distribution of missing data for the primary event are provided in Supplementary Fig. S3*.*

### Lactate plasma levels

Median lactate plasma levels before start of CKRT were 3.2 [1.63–8.7] and 1.5 [1–2.9.9] mmol/L in patients with and without citrate accumulation, respectively. Each one-unit increase in lactate levels on the logarithmic scale (log(mmol/l)) was associated with a corresponding increase in the odds of 2.34 (CI 1.94–2.85, *p* < 0.001) for citrate accumulation. The risk of experiencing citrate accumulation within 14 days ranged from 3.3 (CI 2.06–5.28) % at lactate levels of 0.3 mmol/L to 59.8 (CI 48.88–69.78) % at lactate levels of 25 mmol/L. Figure 1b. represents lactate levels in relation to the probability of citrate accumulation at day 14 after start of CKRT.

### Vasoactive inotropic score (VIS)

The VIS one hour before start of CKRT was 13 [0–33.9.9] points among patients without and 22.5 [1.96–46.1] points among individuals with citrate accumulation. Each 1-unit increase of VIS on the square root scale was associated with an increase of the odds by 1.08 (95%CI 1.03–1.15, *p* = 0.005).

### Parameters of liver injury and impaired systemic perfusion

In patients with and without citrate accumulation, bilirubin plasma levels were 40 [15–163] and 19 [8–48.5.5] µmol/L, FV activities were 28 [15–53] and 64.5 [37–97] %, and INR levels were 1.7 [1.4–2.28] and 1.2 [1.1–1.5], respectively. Citrate accumulation was associated with bilirubin (log(µmol/L): OR 1.44, CI 1.26–1.65, *p* < 0.001), FV (sqrt(%): OR 0.72, CI 0.65–0.79, *p* < 0.001) and INR (log: OR 13.1, CI 7.59–23.2, *p* < 0.001) at the initiation of CKRT. ΔavCO₂ and ScvO_2_ plasma levels were 0.91 kPa [0.59–1.39] and 72.9 [65.1–79.2] % in patients without citrate accumulation and 1.01 [0.62–1.42] kPa and 71.9 [61.7–78.6] % in those with citrate accumulation, respectively. No association was observed with these surrogate markers of impaired global perfusion and citrate accumulation (ΔavCO_2_, sqrt(kPa): OR 0.83, CI 0.34–1.95, *p* = 0.67; ScvO2, %: OR 1.02, CI 1.00 to 1.05, *p* = 0.079).

**Table 2:** Regression models

Variables	Univariable models		Multivariable model	
	OR (95% CI)	*p*-value	OR (95% CI)	*p*-value
Lactate, log(mmol/L)	2.34 (1.94 to 2.85)	**< 0.001**	2.36 (0.94 to 6.40)	0.077
VIS, sqrt	1.08 (1.03 to 1.15)	**0.005**	1.08 (0.86 to 1.35)	0.51
Age, years	1.00 (0.99 to 1.02)	0.62	1.00 (0.96 to 1.05)	0.89
Sex				
F	–		–	
M	0.49 (0.34 to 0.70)	**< 0.001**	0.54 (0.16 to 1.82)	0.31
Bilirubin, log(µmol/L)	1.44 (1.26 to 1.65)	**< 0.001**	1.97 (1.17 to 3.55)	**0.016**
FV, sqrt(%)	0.72 (0.65 to 0.79)	**< 0.001**	0.93 (0.64 to 1.36)	0.71
INR, log	13.1 (7.59 to 23.2)	**< 0.001**	1.92 (0.13 to 28.6)	0.63
Platelets, sqrt(G/L)	0.96 (0.92 to 1.00)	**0.038**	1.13 (0.96 to 1.35)	0.14
ΔavCO_2_, sqrt(kPa)	0.83 (0.34 to 1.95)	0.67	0.78 (0.19 to 3.44)	0.74
ScvO_2_, %	1.02 (1.00 to 1.05)	0.079	0.99 (0.94 to 1.04)	0.7
P/F-ratio	1.00 (1.00 to 1.00)	0.34	1.00 (1.00 to 1.01)	0.65
GCS	1.04 (1.01 to 1.08)	**0.024**	0.95 (0.82 to 1.08)	0.46
Leucocytes, G/L	1.01 (1.00 to 1.02)	0.17	1.03 (0.96 to 1.09)	0.4
CRP, sqrt(mg/dL)	0.92 (0.89 to 0.96)	**< 0.001**	0.90 (0.76 to 1.04)	0.17
pH	0.03 (0.01 to 0.12)	**< 0.001**	15.7 (0.02 to 14,994)	0.41
Creatinine, log(µmol/L)	0.86 (0.66 to 1.13)	0.29	1.04 (0.29 to 3.81)	0.95
Albumin, g/l	1.02 (0.99 to 1.05)	0.2	1.05 (0.96 to 1.15)	0.27
B-DS-ratio, log	2.02 (1.04 to 3.86)	**0.034**	23.9 (1.81 to 575)	**0.029**
Modus				
CVVHD	–		–	
CVVHDF	1.15 (0.80 to 1.66)	0.44	0.08 (0.01 to 0.61)	**0.027**
Post-operative admission				
No			–	
Yes	0.86 (0.60 to 1.21)	0.38	1.03 (0.28 to 3.71)	0.97

The bolded *p*-values indicate all results with *p* < 0.05

OR = Odds ratio, CI = Confidence interval, VIS = Vasoactive inotropic score, FV = Factor V, INR = International normalized ratio, ΔavCO2 = arteriovenous CO_2_ gap, ScvO2 = central vein O_2_ saturation, P/F-ratio = pO2/Fio2 ratio, CRP = C-reactive protein, GCS = Glasgow coma scale, B-DS-ratio = Blood-to dialysate + substitute flow ratio hemodialysis, CVVHDF = Continuous venovenous hemodiafiltration

### Multivariable adjustment

Of the investigated covariables, sex, platelets, GCS, CRP, pH and B-DS-ratio were associated with the primary endpoint in univariable models.

After adjustment in the multivariable model only bilirubin, B-DS-ratio and modality of CKRT were independently associated with citrate accumulation. Results of uni- and multivariable models are provided in Table 2. AUC was 0.72 (CI 0.68–0.76) for the model based solely on lactate levels, 0.58 (CI 0.52–0.63) for the model only including VIS, and 0.89 (CI 0.8–0.99) for the multivariable model (Supplementary Table S4 + Fig. S5). The lactate model displays a significantly improved discriminative performance compared to the VIS model (*p* < 0.001). Table 3 shows the sensitivity, specificity, positive predictive value and negative predictive value for the prediction of citrate accumulation for different lactate plasma levels. Figure S6 in the SI displays the proportion of patients with citrate accumulation above and below specified cut-off thresholds.

**Table 3:** Sensitivity, specificity, positive-, negative predictive values and Youden index

Lactate, mmol/l	Sens	Spec	PPV	NPV	YI
1	0.94	0.24	0.21	0.95	0.18
2.6	0.61	0.72	0.32	0.9	0.33
5	0.41	0.85	0.37	0.87	0.26
10	0.18	0.95	0.45	0.85	0.14
20	0.04	1	0.75	0.83	0.04

The table presents sensitivity (Sens), specificity (Spec), positive predictive values (PPV), negative predictive values (NPV) and Youden Index (YI) for different lactate thresholds

### Longitudinal trajectories of predictors

**Fig. 2:**
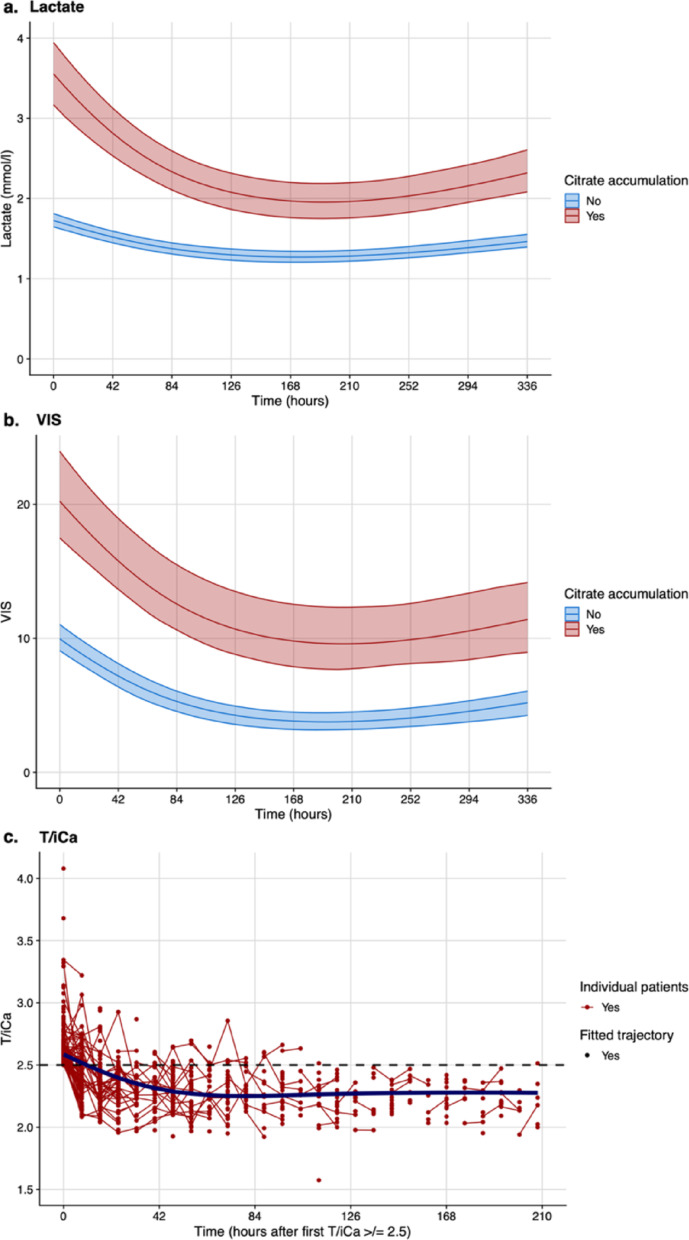
Longitudinal trajectories: Predicted trajectories of lactate (**a**) and the Vasoactive Inotropic Score (VIS, **b**) over time in patients with (red line) and without (blue line) citrate accumulation. The models were calculated on a logarithmic scale for lactate and a square-root scale for VIS, and then back-transformed to their original values for improved readability. The shaded areas represent the 95% confidence intervals. (**c**) Shows individual courses of the total albumin-corrected-to-ionized calcium ratio (T/iCa) among patients after having reached a T/iCa ≥ 2.5 for the first time (Timepoint 0). The fitted (dark violet) line depicts the overall trajectory toward normalization of T/iCa in this patient cohort

Lactate, VIS, bilirubin, FV, INR, platelets, leucocytes, CRP, pH, and B-DS-ratio, but not creatinine, ΔavCO₂, or ScvO₂, showed distinct plasma concentrations at start of CKRT in patients with citrate accumulation compared to those without. Only bilirubin displayed an oppositely directed trajectory in patients with citrate accumulation compared to those without (Fig. 2a/b and Supplementary Table, Fig. S7–9).

### Citrate accumulation and association with severity of disease

Citrate accumulation was associated with ICU mortality (OR 2.67, 95% CI 1.89–3.81, *p* < 0.001) in a univariable model. This association remained significant after adjusting for covariables measured before the initiation of CKRT (OR 3.54, 95% CI 1.55–8.39, *p* = 0.003), but did not persist after adjusting for covariables measured immediately after the start of therapy and at the timepoint of citrate accumulation. Lactate, age, FV, and platelets were the only parameters independently associated with mortality after adjusting for variables measured at the time of citrate accumulation. Citrate accumulation was not associated with the length of ICU stay among survivors (Supplementary Table S10a, b).

### Longitudinal course of T/iCa

Median time from the occurrence of a T/iCa ≥ 2.5 to the discontinuation of RCA-based CKRT was 40 [8–120] h. In 13.2% (21/159) of cases a discontinuation of RCA was conducted during the following 8 h period, in 86.8% (138/159) of cases CKRT was continued with RCA. Discontinuation of RCA was associated with mortality; however, this association did not remain significant after multivariable adjustment (Supplementary Table S11a). Among individuals who discontinued RCA, death occurred in 6 patients (28.57%) within the subsequent 8-hour period after having reached a T/iCa ≥ 2.5.

Among patients with continued RCA, 68.1% (94/138) of cases had a normalization of the T/iCa during further course of CKRT, only 18.8% (26/138) of individuals failed to clear citrate accumulation and in 13.0% (18/138) T/iCa was not quantified again after having reached a level ≥ 2.5. Failed citrate clearance was associated with ICU mortality in the univariable, but not in the multivariable analysis (Supplementary Table S11b).

Among the investigated covariables, lactate was the only predictor independently associated with mortality after multivariable adjustment, indicating that the adverse outcome was driven by the underlying disease reflected in elevated lactate levels, rather than by discontinuation of RCA or failure of citrate clearance. Figure 2c. shows the longitudinal trajectories of the T/iCa among patients after having reached a T/iCa ≥ 2.5. Figure S12 in the SI illustrates the proportions of patients with and without continuation of RCA after reaching a T/iCa ≥ 2.5, the proportions in whom T/iCa normalised or remained pathological, and the reasons for discontinuation of RCA.

## Discussion

This study outlines an incidence proportion of 17% for citrate accumulation using our definition of a T/iCa ≥ 2.5 within 14 days upon initiation of RCA-based CKRT. The study identified an association of citrate accumulation with parameters of liver injury (bilirubin) and CKRT settings (B-DS-ratio and dialysis modality) and confirms lactate plasma levels measured 1 h before the initiation of dialysis as a potential predictor. Furthermore, the study provides evidence that the association between citrate accumulation and mortality is no longer observed after adjusting for covariables of organ failure measured at the time of citrate accumulation.

Assessing the benefits and risks of anticoagulation strategies in CKRT remains a significant challenge in achieving optimal and personalized patient care, as knowledge about the incidence of adverse events, such as citrate accumulation, remains controversial, particularly in patients with systemic shock and liver dysfunction [10, 18, 19].

In the investigated cohort we identified a higher incidence of citrate accumulation compared to previous publications [9, 20]. This may partly be explained by the more stringent criteria for defining citrate accumulation applied by other authors, as well as the longer follow-up period used in our study [9, 18]. As no consensus definition exists for the diagnosis of citrate accumulation, the criteria vary across publications [9, 16]. Notably, studies applying definitions comparable to ours have reported similar incidence levels [21, 22]. Another reason may be our use of albumin-corrected rather than uncorrected total calcium levels to detect citrate accumulation, as applied in most other studies [10]. Albumin correction yields higher total calcium values in hypalbuminaemic patients and thus increases the likelihood of detecting a T/iCa ≥ 2.5. An additional aspect is the retrospective nature of most large studies investigating risk of citrate accumulation, which directly ties the incidence to local policies regarding the use of CKRT in high-risk populations. Our centre encompasses a transplant unit with a large proportion of patients experiencing liver impairment. Additionally, RCA is used as the default anticoagulation strategy even in patients with severe shock and/or liver impairment, which may contribute to an incidence rate closer to that observed in high-risk patients, rather than the average critical care population reported in some works [18]. However, such a high incidence makes our centre ideal for a real-world evaluation of citrate accumulation.

Regarding the timing of citrate accumulation, our results showed the highest incidence at 8 and 16 h after initiation of CKRT, indicating that close monitoring of T/iCa during this period might be essential for the timely detection of patients developing citrate accumulation (Fig. 1a).

While early investigations suggested that impaired liver function was the primary cause of citrate accumulation, recent studies have postulated that mitochondrial dysfunction due to circulatory shock and impaired tissue perfusion, might be a key contributing factor [7]. In our cohort, several parameters indicative of liver injury, including FV, INR, and bilirubin, were altered in patients experiencing citrate accumulation. Notably, bilirubin was the only parameter reflecting organ dysfunction that remained significant after multivariable adjustment and the only one that showed a contrasting trajectory in its longitudinal course compared to patients without citrate accumulation. Moreover, impairment of global perfusion, surrogately assessed via ScvO2 and ΔavCO₂, was not apparently associated with citrate accumulation. These findings highlight impaired hepatic clearance as the probable key determinant of citrate accumulation in this cohort.

In addition to bilirubin, parameters related to filter settings, such as the B-DS-ratio and dialytic (compared to convective) filtration modes, demonstrated an association with citrate accumulation. The B-DS-ratio could be considered as factor influencing citrate net overload, rather than insufficient citrate metabolism, since an increase in the B–DS ratio results in a higher net citrate administration to the patient [7]. The potential increased risk of citrate accumulation with CVVHD compared to CVVHDF remains to be determined. However, it may be related to differences in citrate clearance, as CVVHDF can maintain a consistent ultrafiltration component even when filter patency declines over time [23, 24].

While citrate overload has been recognized to exert potential toxic effects, its effective impact on patient outcomes has not been determined sufficiently. Link and colleagues showed an association between the T/iCa and 28-day mortality [25]. While our analysis confirmed an independent association of a T/iCa ≥ 2.5 with ICU-mortality when adjusted for covariables measured before start of CKRT, an adjustment with factors measured at timepoint of citrate accumulation could not confirm this association. Since covariables measured before the initiation of therapy may have introduced time-dependent bias, the data cannot ascertain whether citrate accumulation resulting from the initiation of CKRT contributes to the adverse outcome, or if citrate accumulation and mortality occur independently as part of the natural progression of the disease. Given these findings, the true relationship between T/iCa and fatal outcomes remains uncertain, highlighting the need for further investigations.

Interestingly, our analysis showed that discontinuation of RCA after reaching the criteria for citrate accumulation was also associated with a higher risk of mortality. This association is, however, most likely driven by confounding by indication and reverse causation: patients with greater morbidity were more likely to have RCA completely discontinued, often in the context of death, whereas patients with less severe illness were more often maintained on adjusted RCA-based CKRT despite citrate accumulation.

For the early identification of patients at risk of citrate accumulation, lactate has been proposed as a potential predictor [7, 19]. Khadzhynov and colleagues demonstrated higher baseline lactate levels in patients who experienced citrate accumulation within the first 48 h after the onset of CKRT, identifying an optimal cut-off value of 2.39 mmol/L [9]. In line with this work, our investigation yielded similar results over an extended follow-up period of 14 days, identifying an optimal cut-off value of 2.6 mmol/L, as determined by the Youden Index. However, this cut-off value is associated with low sensitivity (61%) and moderate specificity (72%), raising concerns about its practicality. In contrast, the probability estimation provided in this study demonstrated reliable accuracy for lactate in identifying citrate accumulation, as indicated by the AUC. In contrast to the work of Khadzhynov and colleagues, we focused solely on baseline lactate levels for the prediction model and reported dynamic changes descriptively, as our primary aim was to identify the unbiased association of lactate levels with the outcome prior to the initiation of CKRT [9].

Originally regarded solely as a by-product of anaerobic metabolism, lactate is now understood to also serve as a mitochondrial substrate and signalling molecule [26]. As both lactate and citrate are metabolised within mitochondria, impaired mitochondrial function provides a common mechanism for their accumulation and explains the association between these parameters observed in this study. As outlined previously, in our cohort we observed a strong association of citrate accumulation with parameters of liver injury rather than with systemic mitochondrial dysfunction from circulatory shock and impaired tissue perfusion. Accordingly, lactate in this patient collective may primarily reflect impaired hepatic mitochondrial clearance rather than systemic dysfunction due to shock.

While the study’s strengths lie in its large sample size and the high resolution of clinical and laboratory parameters, several limitations must be considered. Although the investigated centre uses RCA as the default anticoagulation strategy, even in patients at elevated risk for citrate accumulation, 173 of the screened patients did not receive RCA. In an additional 58 cases, CKRT was initiated with heparin and later switched to RCA. Although these patients were excluded from the analysis, their deviation from our institutional standard protocols may have introduced selection bias.

An additional limitation may be the presence of unmeasured confounding factors influencing citrate metabolism, such as propofol, which can impair mitochondrial function and potentially affect citrate metabolism in a dose-dependent manner [27].

While the presented results show predicted probabilities for citrate accumulation based on the original dataset, the study does not include either an internal or external validation cohort. Therefore, further investigations are needed to validate these algorithms before broader conclusions can be drawn for a larger patient population.

## Conclusion

In summary, this study reveals a high incidence of citrate accumulation in a tertiary university centre and identifies bilirubin, B-DS-ratio, and CKRT modalities as associated factors, suggesting that failed hepatic clearance and excessive citrate administration are key underlying mechanisms. Furthermore, the study confirms an association between lactate levels measured before CKRT initiation and citrate accumulation, highlighting lactate as a potential predictor for personalized risk stratification. However, the impact of citrate accumulation on patient outcomes remains uncertain and requires further investigations.

## Supplementary Information

Supplementary Material 1

## Data Availability

The datasets used and/or analysed during the current study are available from the corresponding author on reasonable request.

## Abbreviations

ΔavCO₂: Arteriovenous CO₂ gap
AUC: Area under the receiver operating characteristic curve
B-DS-ratio: Blood-to-dialysate + substitute flow ratio
CI: 95% confidence interval
CRP: C-reactive protein
CKRT: Continuous kidney replacement therapy
CVVHD: Continuous venovenous hemodialysis
CVVHDF: Continuous venovenous hemodiafiltration
EPR: Electronic patient record
FV: Factor V
GCS: Glasgow coma scale
ICU: Intensive care unit
INR: International normalized ratio
IQR: Interquartile ranges
KDIGO: Kidney Disease: Improving Global Outcomes
OR: Odds ratio
P/F-ratio: pO2/Fio2 ratio
RCA: Regional citrate anticoagulation
KRT: Kidney replacement therapy
ScvO₂: Central venous oxygen saturation
STROBE: Strengthening the Reporting of Observational Studies in Epidemiology
T/iCa: Total albumin-corrected-to-ionized calcium ratio
VIS: Vasoactive inotropic score
