# Supplementary material for: Incidence, severity, and predictors of citrate accumulation during continuous kidney replacement therapy in the critically ill
Source: Crit Care. 2025 Nov 3;29:468. doi: 10.1186/s13054-025-05691-2 (PMC12581596; doi:10.1186/s13054-025-05691-2)
Supplement: Supplementary file 1 — Supplementary Material 1 [file 13054_2025_5691_MOESM1_ESM.docx]

**Supplemental Information:**

**Incidence, severity, and predictors of citrate accumulation during continuous kidney replacement therapy in the critically ill.**

Mattia M. Müller^1^, Alexa Weber^2^, Jan Bartussek^1,3^, Jasmin Bachmann^2^, Alix Buhlmann^2^, Gabor Kadler^2^, Caroline John^2^, Rolf Erlebach^1^, Daniel A. Hofmaenner^2^, Rea Andermatt^2^, Marc Thorsten Schmidt^2^, Reto A. Schuepbach^1^, Christoph C. Ganter^2^, Pedro D. Wendel-Garcia^2,4^*, Sascha David^1,5^*

1. Institute of Intensive Care Medicine, University Hospital and University of Zurich, Zurich, Switzerland
2. Institute of Intensive Care Medicine, University Hospital Zurich, Zurich, Switzerland
3. Department for Quantitative Biomedicine, University of Zurich, Zurich, Switzerland
4. Division of Cardiothoracic Anaesthesia and Intensive Care Medicine, Department of Anaesthesiology, General Intensive Care and Pain Medicine, Medical University Vienna and Vienna General Hospital, Vienna, Austria
5. Department of Nephrology and Hypertension, Medical School Hannover, Hannover, Germany

* SD and PDWG contributed equally to this study as last authors

Corresponding author: [sascha.david@usz.ch](mailto:sascha.david@usz.ch)

| **Weight (kg)** | **50** | **60** | **70** | **80** | **90** | **100** |
| --- | --- | --- | --- | --- | --- | --- |
| Blood flow [mL/min] | 100 | 120 | 120 | 120 | 120 | 120 |
| Substituate post filter [mL/h] | 250 | 250 | 250 | 500 | 500 | 500 |
| Dialysate [mL/h] | 500 | 700 | 900 | 1000 | 1200 | 1300 |
| **PBP (mL/h)** |  |  |  |  |  |  |
| Citrate 2.5 mmol/L blood | 833 | 1000 | 1000 | 1000 | 1000 | 1000 |
| Citrate 3 mmol/L blood | 1000 | 1200 | 1200 | 1200 | 1200 | 1200 |
| Citrate 3.5 mmol/L blood | 1167 | 1400 | 1400 | 1400 | 1400 | 1400 |
| Ca++-Substitution in % of the filtrated Ca++ | 100 | 100 | 100 | 100 | 100 | 100 |
| **CKRT dose (ml/kg KG/h)** |  |  |  |  |  |  |
| Citrate 2.5 mmol/L blood | 32 | 33 | 31 | 31 | 30 | 28 |
| Citrate 3 mmol/L blood | 35 | 36 | 34 | 34 | 32 | 30 |
| Citrat 3.5 mmol/L blood | 38 | 39 | 36 | 36 | 34 | 32 |
|  |  |  |  |  |  |  |
| **Targeted postfilter iCa** | 0.25 – 0.5 mmol/L | |  |  |  |  |

**Tbl. S1 a. - Protocol start settings - Prismaflex System (Baxter, Deerfield, USA)**

The table presents weight-adapted initial settings for continuous kidney replacement therapy (CKRT) using the Prismaflex System, including blood flow, dialysate flow, and the pre-blood pump (PBP) flow rate for citrate administration.

| **Citrate-associated acid-base disturbances** | |
| --- | --- |
| Metabolic Alkalosis (pH > 7.45) | Gradually increase dialysate flow rate up to a maximum **2500 mL/h** |
| Metabolic Acidosis (pH < 7.3) | Gradually decrease dialysate flow rate down to a minimum of **500 mL/h** |
|  | |
| **Non-citrate-associated severe metabolic acidosis** | |
| Persistent pH < 7.3 with High Catecholamine Demand (e.g., Sepsis) | Gradually increase post-filter substitution flow up to 2000 mL/h |

**Tbl. S1 b. - Protocol management acid-base balance - Prismaflex System**

The table presents the local protocol for adjusting kidney replacement device settings with the Prismaflex System to manage both citrate-related and unrelated metabolic disturbances.

| **Targeted CKRT dose** |  | **Body weight** | (kg) |  |  |  |  |  |  |  |
| --- | --- | --- | --- | --- | --- | --- | --- | --- | --- | --- |
|  |  | 50 | 60 | 70 | 80 | 90 | 100 | 110 | 120 | 130 |
| **20 ml/kg/h** | **Dialysate (ml/h)** | 1000 | 1200 | 1400 | 1600 | 1800 | 2000 | 2100 | 2200 | 2300 |
|  | **Blood flow (ml/min)** | 50 | 60 | 70 | 80 | 90 | 100 | 100 | 110 | 110 |
| **25 ml/kg/h** | **Dialysat (ml)** | 1250 | 1500 | 1750 | 2000 | 2250 | 2500 | 2600 | 2700 | 2800 |
|  | **Blood flow (ml/min)** | 70 | 80 | 90 | 100 | 110 | 120 | 130 | 140 | 150 |
| **30 ml/kg/h** | **Dialysat (ml)** | 1500 | 1800 | 2100 | 2400 | 2700 | 3000 | 3100 | 3200 | 3300 |
|  | **Blood flow (ml/min)** | 80 | 90 | 110 | 120 | 130 | 150 | 160 | 160 | 170 |
|  |  |  |  |  |  |  |  |  |  |  |
| **Targeted postfilter iCa** | 0.25 – 0.34 mmol/L |  |  |  |  |  |  |  |  |  |

**Tbl. S1 c. - Protocol start settings - multiFiltrate (Fresenius Medical Care, Bad Homburg, Germany)**

The table presents initial settings for continuous kidney replacement therapy (CKRT) with the multiFiltrate device, tailored to different target dialysis doses and adjusted for individual body weight.

| **Acid-base disturbances** | |
| --- | --- |
| Metabolic Alkalosis | ↓ Blood Flow ± ↑ Dialysate Flow Rate |
| Metabolic Acidosis | ↑ Blood Flow ± ↓ Dialysate Flow Rate |

**Tbl. S1 d. - Protocol management acid-base balance - multiFiltrate**

The table presents the local protocol for adjusting multiFiltrate device settings in response to acid-base disturbances.


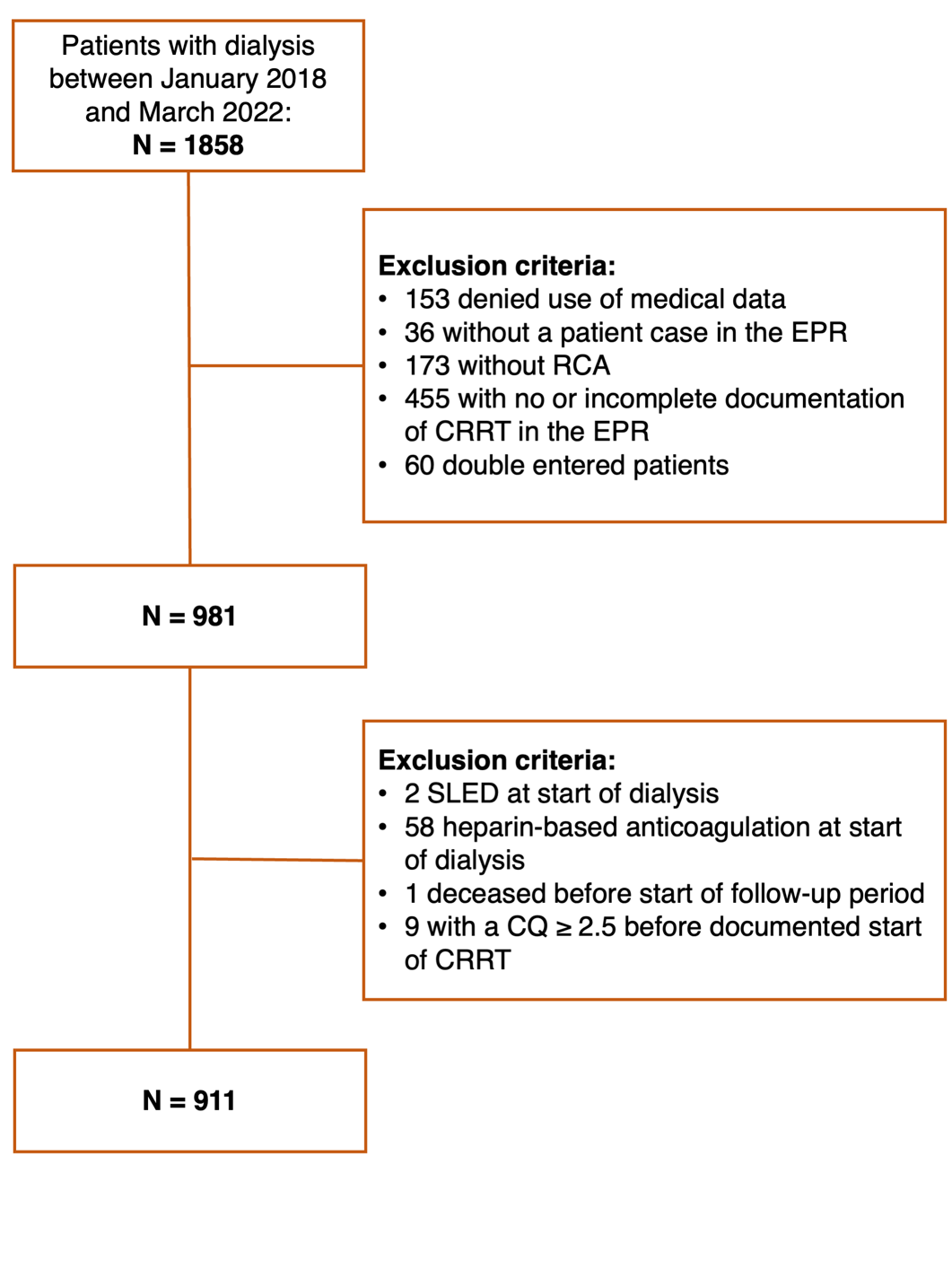


**Fig. S2 - Screening and inclusion process**

Out of 1,858 patients who received CKRT between January 2018 and March 2022, 911 met the criteria for inclusion in the final analysis.

**Fig. S3 - Competing events and missing data**

The plot shows the cumulative incidence for citrate accumulation (solid line) and competing events such as discharge from the ICU with transfer to another hospital/ICU facility (dashed line), discontinuation of RCA-based CKRT (dotted line), or death (dash-dotted line), along with the corresponding 95% confidence intervals. The legend displays the number of patients at risk, events, and missing values for the calculation of the primary event of citrate accumulation among individuals at risk.

| **Tbl. S4 - Area under the curve (AUC) of models** | | |
| --- | --- | --- |
| **Model** | **AUC** | **95% CI** |
| Lactate | 0.72 | 0.68 - 0.76 |
| VIS | 0.58 | 0.52 - 0.63 |
| Sex | 0.58 | 0.54 - 0.62 |
| Bilirubin | 0.64 | 0.59 - 0.69 |
| FV | 0.73 | 0.68 - 0.78 |
| INR | 0.76 | 0.72 - 0.81 |
| Platelets | 0.55 | 0.5 - 0.6 |
| GCS | 0.56 | 0.5 - 0.61 |
| CRP | 0.63 | 0.58 - 0.68 |
| pH | 0.6 | 0.55 - 0.66 |
| B-DS-ratio | 0.54 | 0.48 - 0.6 |
| Multivariable | 0.89 | 0.8 - 0.99 |

95% CI = 95 % confidence interval, VIS = Vasoactive inotropic score, FV = Factor V, INR = International normalized ratio, CRP = C-reactive protein, B-DS-ratio = Blood-to-dialysate+substituate flow ratio


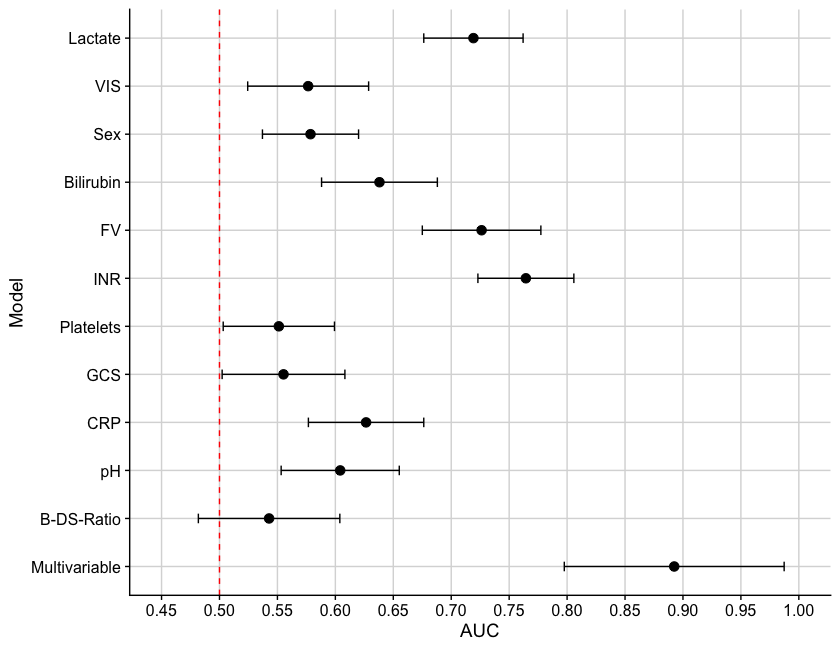


**Fig. S5 - Area under the curve (AUC)**

The figure presents the AUC for the various univariable and multivariable models, along with 95%

confidence intervals. VIS = Vasoactive Inotropic Score, FV = Factor V, INR = International normalized ratio, CRP = C-reactive protein, B-DS Ratio = Blood-to-dialysate+substituate flow ratio.

**Fig. S6 - Lactate thresholds**

The figure shows the percentage of patients with (red) and without (blue) citrate accumulation, above and below lactate thresholds of 2.6 mmol/L (a), 5 mmol/L (b), 10 mmol/L (c), and 20 mmol/L (d).

| **Tbl. S7 - Mixed-effects models for lactate and VIS** | | | | | | |
| --- | --- | --- | --- | --- | --- | --- |
|  | **Lactate** | | | **VIS** | | |
| *Predictors* | *Estimates* | *95% CI* | *p-value* | *Estimates* | *95% CI* | *p-value* |
| Intercept | 0.55 | 0.50 – 0.60 | **<0.001** | 3.17 | 2.99 – 3.35 | **<0.001** |
| Time (1st degree) | -0.55 | -0.59 – -0.50 | **<0.001** | -2.18 | -2.36 – -2.01 | **<0.001** |
| Time (2nd degree) | -0.03 | -0.07 – 0.01 | 0.141 | -0.38 | -0.52 – -0.24 | **<0.001** |
| Citrate accumulation | 0.72 | 0.60 – 0.84 | **<0.001** | 1.34 | 0.91 – 1.78 | **<0.001** |
| Time (1st degree)xCitrate accumulation | -0.52 | -0.64 – -0.40 | **<0.001** | -0.3 | -0.74 – 0.14 | 0.180 |
| Time (2nd degree)xCitrate accumulation | -0.12 | -0.2 – -0.04 | **0.003** | -0.16 | -0.47 – 0.14 | 0.295 |

95 % CI = 95% Confidence interval, VIS = Vasoactive inotropic score

| **Tbl. S8 a. - Mixed-effects models for covariables** | | | | | | |
| --- | --- | --- | --- | --- | --- | --- |
|  | **Bilirubin, log(µmol/L)** | | | **Factor V, sqrt(%)** | | |
| *Predictors* | *Estimates* | *95% CI* | *p-value* | *Estimates* | *95% CI* | *p-value* |
| Intercept | 3.24 | 3.14 – 3.34 | **<0.001** | 7.72 | 7.45 – 7.99 | **<0.001** |
| Time (1st degree) | 0.25 | 0.18 – 0.33 | **<0.001** | 1.94 | 1.57 – 2.30 | **<0.001** |
| Time (2nd degree) | 0.34 | 0.24 – 0.44 | **<0.001** | 3.07 | 2.66 – 3.47 | **<0.001** |
| Time (3rd degree) | 0.13 | 0.06 – 0.21 | **<0.001** | 1.37 | 0.96 – 1.78 | **<0.001** |
| Citrate accumulation | 0.74 | 0.51 – 0.97 | **<0.001** | -2.38 | -2.91 – -1.85 | **<0.001** |
| Time (1st degree)xCitrate accumulation | 0.02 | -0.15 – 0.19 | 0.788 | 0.56 | -0.09 – 1.22 | 0.092 |
| Time (2nd degree)xCitrate accumulation | 0.34 | 0.12 – 0.56 | **0.002** | 0.26 | -0.47 – 0.99 | 0.488 |
| Time (3rd degree)xCitrate accumulation | 0.23 | 0.08 – 0.39 | **0.002** | 1.09 | 0.46 – 1.72 | **0.001** |
|  |  |  |  |  |  |  |
|  | **INR, log** | | | **Platelets, sqrt (G/L)** | | |
| *Predictors* | *Estimates* | *95% CI* | *p-value* | *Estimates* | *95% CI* | *p-value* |
| Intercept | 0.31 | 0.29 – 0.33 | **<0.001** | 11.21 | 10.90 – 11.52 | **<0.001** |
| Time (1st degree) | -0.11 | -0.13 – -0.09 | **<0.001** | 1.17 | 0.85 – 1.48 | **<0.001** |
| Time (2nd degree) | -0.27 | -0.30 – -0.24 | **<0.001** | 0.74 | 0.35 – 1.13 | **<0.001** |
| Time (3rd degree) | -0.09 | -0.11 – -0.06 | **<0.001** | 2.6 | 2.29 – 2.90 | **<0.001** |
| Citrate accumulation | 0.32 | 0.28 – 0.37 | **<0.001** | -0.94 | -1.66 – -0.22 | **0.01** |
| Time (1st degree)xCitrate accumulation | -0.11 | -0.16 – -0.05 | **<0.001** | -1.78 | -2.49 – -1.07 | **<0.001** |
| Time (2nd degree)xCitrate accumulation | -0.22 | -0.28 – -0.15 | **<0.001** | -2.22 | -3.10 – -1.34 | **<0.001** |
| Time (3rd degree)xCitrate accumulation | -0.18 | -0.23 – -0.14 | **<0.001** | -0.8 | -1.44 – -0.17 | **0.013** |
|  |  |  |  |  |  |  |
|  | **ΔavCO_2_, sqrt(kPa)** | | | **ScvO2, %** | | |
| *Predictors* | *Estimates* | *95% CI* | *p-value* | *Estimates* | *95% CI* | *p-value* |
| Intercept | 0.92 | 0.91 – 0.94 | **<0.001** | 69.46 | 68.52 – 70.39 | **<0.001** |
| Time (1st degree) | 0.11 | 0.06 – 0.15 | **<0.001** | -5.2 | -6.71 – -3.69 | **<0.001** |
| Time (2nd degree) |  |  |  | -10.34 | -12.19 – -8.49 | **<0.001** |
| Time (3rd degree) |  |  |  | -4.51 | -6.06 – -2.95 | **<0.001** |
| Citrate accumulation | -0.01 | -0.05 – 0.02 | 0.457 | 1.15 | -0.98 – 3.27 | 0.29 |
| Time (1st degree)xCitrate accumulation | -0.08 | -0.17 – 0.02 | 0.114 | -0.38 | -3.58 – 2.82 | 0.816 |
| Time (2nd degree)xCitrate accumulation |  |  |  | 1.75 | -2.28 – 5.78 | 0.395 |
| Time (3rd degree)xCitrate accumulation |  |  |  | -1.5 | -4.39 – 1.39 | 0.308 |

95% CI = 95 % confidence interval, VIS = Vasoactive inotropic score, FV = Factor V, INR = International normalized ratio, CRP = C-reactive protein, ΔavCO_2_ = arteriovenous CO_2_ difference

| **Tbl. S8 b. - Mixed-effects models for covariables** | | | | | | |
| --- | --- | --- | --- | --- | --- | --- |
|  | **Leucocytes, sqrt(G/L)** | | | **CRP, sqrt(mg/dL)** | | |
| *Predictors* | *Estimates* | *95% CI* | *p-value* | *Estimates* | *95% CI* | *p-value* |
| Intercept | 3.65 | 3.55 – 3.74 | **<0.001** | 11.42 | 11.06 – 11.77 | **<0.001** |
| Time (1st degree) | 0.34 | 0.23 – 0.44 | **<0.001** | -1.53 | -1.98 – -1.08 | **<0.001** |
| Time (2nd degree) | -0.1 | -0.23 – 0.03 | 0.124 | -1.08 | -1.66 – -0.49 | **<0.001** |
| Time (3rd degree) | -0.26 | -0.36 – -0.16 | **<0.001** | -1.86 | -2.30 – -1.42 | **<0.001** |
| Citrate accumulation | 0.3 | 0.08 – 0.52 | **0.008** | -2.84 | -3.67 – -2.01 | **<0.001** |
| Time (1st degree)xCitrate accumulation | -0.05 | -0.29 – 0.20 | 0.711 | -0.76 | -1.83 – 0.32 | 0.168 |
| Time (2nd degree)xCitrate accumulation | -0.32 | -0.62 – -0.02 | **0.037** | 1.66 | 0.27 – 3.04 | **0.019** |
| Time (3rd degree)xCitrate accumulation | -0.29 | -0.50 – -0.07 | **0.009** | 0.97 | 0.03 – 1.90 | **0.044** |
|  |  |  |  |  |  |  |
|  | **pH** | | | **Creatinine, log(µmol/L)** | | |
| *Predictors* | *Estimates* | *95% CI* | *p-value* | *Estimates* | *95% CI* | *p-value* |
| Intercept | 7.35 | 7.35 – 7.36 | **<0.001** | 5.26 | 5.23 – 5.30 | **<0.001** |
| Time (1st degree) | 0.02 | 0.01 – 0.02 | **<0.001** | -0.23 | -0.27 – -0.19 | **<0.001** |
| Time (2nd degree) | 0.14 | 0.13 – 0.14 | **<0.001** | -1.12 | -1.17 – -1.07 | **<0.001** |
| Time (3rd degree) | 0.04 | 0.04 – 0.05 | **<0.001** | -0.36 | -0.40 – -0.32 | **<0.001** |
| Citrate accumulation | -0.05 | -0.07 – -0.04 | **<0.001** | -0.05 | -0.14 – 0.04 | 0.241 |
| Time (1st degree)xCitrate accumulation | 0.03 | 0.01 – 0.04 | **<0.001** | 0.02 | -0.08 – 0.11 | 0.75 |
| Time (2nd degree)xCitrate accumulation | 0.04 | 0.03 – 0.06 | **<0.001** | 0.11 | -0.02 – 0.23 | 0.086 |
| Time (3rd degree)xCitrate accumulation | 0.02 | 0.01 – 0.04 | **<0.001** | -0.05 | -0.13 – 0.03 | 0.248 |
|  |  |  |  |  |  |  |
|  | **B-DS-ratio** | | |  | | |
| *Predictors* | *Estimates* | *95% CI* | *p-value* |  |  |  |
| Intercept | 1.24 | 1.21 – 1.26 | **<0.001** |  |  |  |
| Time (1st degree) | -0.06 | -0.07 – -0.04 | **<0.001** |  |  |  |
| Time (2nd degree) | -0.13 | -0.15 – -0.11 | **<0.001** |  |  |  |
| Time (3rd degree) | -0.05 | -0.07 – -0.03 | **<0.001** |  |  |  |
| Citrate accumulation | 0.07 | 0.01 – 0.13 | **0.015** |  |  |  |
| Time (1st degree)xCitrate accumulation | -0.03 | -0.08 – 0.01 | 0.178 |  |  |  |
| Time (2nd degree)xCitrate accumulation | -0.18 | -0.24 – -0.13 | **<0.001** |  |  |  |
| Time (3rd degree)xCitrate accumulation | -0.09 | -0.13 – -0.05 | **<0.001** | - |  |  |

95% CI = 95 % confidence interval, B-DS-ratio = Blood-to-dialysate+substituate flow ratio

**Fig. S9 - Longitudinal trajectories**

The graph displays predicted estimates and 95% confidence intervals over time for covariates among patients with (red) and without (blue) citrate accumulation. VIS = Vasoactive Inotropic Score, INR = International normalized ratio, ScvO_2_ = central venous O2 saturation, CRP = C-reactive protein, B-DS-ratio = Blood-to-dialysate+substituate flow ratio.

| **Table S10 a. - Association of citrate accumulation with ICU mortality** | | | | | | | | |
| --- | --- | --- | --- | --- | --- | --- | --- | --- |
| **Characteristics** | **Citrate accumulation univ.** | | **Before start CKRT** | | **After start CKRT** | | **At timepoint T/iCa ≥ 2.5** | |
|  | **OR** **(95% CI)***^1^* | **p-value** | **OR** **(95% CI)***^1^* | **p-value** | **OR** **(95% CI)***^1^* | **p-value** | OR (95% CI)^1^ | p-value |
| Citrate accumulation |  |  |  |  |  |  |  |  |
| No | — |  | — |  | — |  |  |  |
| Yes | 2.67 (1.89 to 3.81) | **<0.001** | 3.54 (1.55 to 8.39) | **0.003** | 3.56 (0.42 to 42.8) | 0.27 | 3.98 (0.82 to 22.0) | 0.094 |
| Lactate, log(mmol/L) |  |  | 1.74 (1.05 to 2.96) | **0.035** | 3.35 (0.74 to 18.7) | 0.13 | 9.11 (2.26 to 52.5) | **0.005** |
| VIS, sqrt |  |  | 1.14 (1.00 to 1.31) | 0.057 | 1.40 (1.00 to 2.16) | 0.087 | 0.97 (0.69 to 1.32) | 0.85 |
| Age, years |  |  | 1.06 (1.03 to 1.09) | **<0.001** | 1.13 (1.04 to 1.26) | **0.009** | 1.11 (1.04 to 1.20) | **0.005** |
| Sex |  |  |  |  |  |  |  |  |
| F |  |  | — |  | — |  | — |  |
| M |  |  | 1.07 (0.48 to 2.41) | 0.87 | 2.41 (0.41 to 16.8) | 0.34 | 0.78 (0.15 to 4.44) | 0.77 |
| Bilirubin, log(µmol/L) |  |  | 1.06 (0.77 to 1.46) | 0.71 | 2.02 (0.86 to 5.51) | 0.13 | 2.09 (1.00 to 4.88) | 0.064 |
| Factor V, sqrt(%) |  |  | 1.09 (0.88 to 1.34) | 0.43 | 0.75 (0.41 to 1.26) | 0.31 | 2.21 (1.27 to 4.32) | **0.01** |
| INR, log |  |  | 0.69 (0.13 to 3.30) | 0.64 | 0.01 (0.00 to 1.20) | 0.12 | 0.31 (0.00 to 12.4) | 0.55 |
| Platelets, sqrt(G/L) |  |  | 0.86 (0.77 to 0.95) | **0.004** | 0.73 (0.50 to 0.98) | 0.058 | 0.67 (0.47 to 0.88) | **0.011** |
| GCS |  |  | 0.92 (0.85 to 1.00) | **0.043** | 0.92 (0.77 to 1.08) | 0.31 | 0.87 (0.71 to 1.05) | 0.16 |
| Leucocytes, G/L |  |  | 1.01 (0.97 to 1.05) | 0.67 | 0.99 (0.92 to 1.11) | 0.85 | 1.07 (0.99 to 1.16) | 0.11 |
| CRP, sqrt(mg/dL) |  |  | 0.99 (0.92 to 1.08) | 0.88 | 1.05 (0.85 to 1.32) | 0.67 | 0.98 (0.84 to 1.15) | 0.82 |
| pH |  |  | 0.49 (0.02 to 7.80) | 0.63 | 0.00 (0.00 to 20.0) | 0.24 | 0.00 (0.00 to 14.5) | 0.19 |
| Creatinine, log(µmol/L) |  |  | 0.81 (0.43 to 1.53) | 0.52 | 2.62 (0.50 to 16.7) | 0.27 | 4.52 (0.67 to 38.2) | 0.14 |
| B-DS-ratio, log |  |  | 0.42 (0.08 to 2.08) | 0.29 | 0.80 (0.01 to 51.7) | 0.91 | 3.03 (0.12 to 95.1) | 0.5 |
| Modus |  |  |  |  |  |  |  |  |
| CVVHD |  |  | — |  | — |  | — |  |
| CVVHDF |  |  | 2.24 (0.79 to 6.65) | 0.14 | 34.4 (1.70 to 1,593) | **0.036** | 1.46 (0.16 to 14.0) | 0.73 |
| Post-Operative Admission |  |  |  |  |  |  |  |  |
| No |  |  | — |  | — |  | — |  |
| Yes |  |  | 0.71 (0.34 to 1.46) | 0.35 | 0.31 (0.04 to 1.87) | 0.23 | 2.82 (0.59 to 16.5) | 0.21 |

The table presents the association between citrate accumulation and mortality, analyzed using a univariable model (univ.) and after adjusting for covariables measured at different time points: prior to the initiation of CKRT (Before start CKRT), immediately after the start of CKRT (After start CKRT), and at the time of citrate accumulation occurrence (At timepoint T/iCa ≥ 2.5). In individuals without citrate accumulation, covariables measured 8 h after the initiation of CKRT were used, corresponding to the average time of citrate accumulation occurrence. OR = Odds ratio, 95% CI = 95 % confidence interval, VIS = Vasoactive Inotropic Score, INR = International normalized ratio, CRP = C-reactive protein, B-DS-ratio = Blood-to-dialysate+substituate flow ratio, GCS = Glasgow coma scale, CVVHD = Continuous venonenous hemodialysis, CVVHDF = Continuous venovenous hemofiltration.

| **Table S10 b. - Association of citrate accumulation with length of ICU stay in survivors** | | | | | | | | |
| --- | --- | --- | --- | --- | --- | --- | --- | --- |
| **Characteristics** | **Citrate accumulation univ.** | | **Before start CKRT** | | **After start CKRT** | | **At timepoint T/iCa ≥ 2.5** | |
|  | **exp (Beta) (95% CI)***^1^* | **p-value** | **exp (Beta) (95% CI)***^1^* | **p-value** | **exp (Beta) (95% CI)***^1^* | **p-value** | **exp (Beta) (95% CI)***^1^* | **p-value** |
| Citrate accumulation |  |  |  |  |  |  |  |  |
| No | — |  | — |  | — |  |  |  |
| Yes | 1.15 (0.86 to 1.60) | 0.37 | 1.03 (0.62 to 1.76) | 0.92 | 1.19 (0.58 to 2.59) | 0.64 | 1.03 (0.62 to 1.74) | 0.91 |
| Lactate, log(mmol/L) |  |  | 1.42 (1.10 to 1.84) | **0.011** | 1.13 (0.77 to 1.65) | 0.5 | 1.21 (0.83 to 1.74) | 0.36 |
| VIS, sqrt |  |  | 1.06 (0.98 to 1.14) | 0.14 | 1.03 (0.92 to 1.17) | 0.53 | 1.05 (0.97 to 1.15) | 0.21 |
| Age, years |  |  | 0.98 (0.97 to 1.00) | **0.02** | 0.99 (0.97 to 1.01) | 0.49 | 0.99 (0.97 to 1.01) | 0.14 |
| Sex |  |  |  |  |  |  |  |  |
| F |  |  | — |  | — |  | — |  |
| M |  |  | 1.48 (0.91 to 2.36) | 0.078 | 1.40 (0.69 to 2.79) | 0.29 | 1.44 (0.89 to 2.28) | 0.14 |
| Bilirubin, log(µmol/L) |  |  | 1.00 (0.87 to 1.15) | 0.98 | 0.85 (0.69 to 1.05) | 0.12 | 1.02 (0.79 to 1.33) | 0.84 |
| Factor V, sqrt(%) |  |  | 1.11 (1.00 to 1.22) | **0.041** | 0.95 (0.85 to 1.07) | 0.41 | 1.18 (1.04 to 1.34) | **0.015** |
| INR, log |  |  | 0.77 (0.31 to 2.02) | 0.55 | 0.70 (0.26 to 2.02) | 0.5 | 0.90 (0.31 to 2.80) | 0.85 |
| Platelets, sqrt(G/L) |  |  | 0.97 (0.93 to 1.02) | 0.24 | 0.99 (0.91 to 1.07) | 0.68 | 0.95 (0.89 to 1.02) | 0.1 |
| B-DS-ratio, log |  |  | 0.56 (0.25 to 1.29) | 0.2 | 0.37 (0.09 to 1.52) | 0.15 | 0.55 (0.25 to 1.23) | 0.19 |
| Modus |  |  |  |  |  |  |  |  |
| CVVHD |  |  | — |  | — |  | — |  |
| CVVHDF |  |  | 1.34 (0.77 to 2.39) | 0.34 | 2.01 (0.85 to 5.05) | 0.16 | 1.65 (0.92 to 3.03) | 0.13 |

The table shows the association between citrate accumulation and length of ICU stay in ICU survivors. Analysis was conducted in an univariable model (univ.) and after adjusting for covariables measured prior to the initiation of CKRT (Before start CKRT), immediately after the start of CKRT (After start CKRT), and at the time of citrate accumulation occurrence (At timepoint T/iCa ≥ 2.5). 95% CI = 95 % confidence interval, VIS = Vasoactive Inotropic Score, INR = International normalized ratio, B-DS-ratio = Blood-to-dialysate+substituate flow ratio, CVVHD = Continuous venonenous hemodialysis, CVVHDF = Continuous venovenous hemofiltration.

| **Table S11 a. - Continuation vs. discontinuation of CKRT after citrate accumulation  and association with ICU mortality** | | | | | | | |
| --- | --- | --- | --- | --- | --- | --- | --- |
| **Characteristics** | | **Univariable** | | | | **Multivariable** | |
|  | | **OR**  **(95% CI)** | | | **p-value** | **OR**  **(95% CI)** | **p-value** |
| Continuation of CKRT | |  | | |  |  |  |
| No | | — | | |  | — |  |
| Yes | | 0.21 (0.05 to 0.65) | | | **0.015** | 0.60 (0.02 to 9.70) | 0.73 |
| Factor V, sqrt(%) | |  | | |  | 1.16 (0.66 to 2.03) | 0.61 |
| Bilirubin, log(µmol/L) | |  | | |  | 1.14 (0.60 to 2.16) | 0.68 |
| INR, log | |  | | |  | 0.35 (0.01 to 11.5) | 0.57 |
| Platelets, sqrt(G/L) | |  | | |  | 0.84 (0.67 to 1.03) | 0.11 |
| Lactate, log(mmol/L) | |  | | |  | 4.90 (1.61 to 18.2) | **0.009** |
| VIS, sqrt | |  | | |  | 1.10 (0.88 to 1.41) | 0.42 |
| Age, years | |  | | |  | 1.02 (0.98 to 1.07) | 0.31 |
| Modus | |  | | |  |  |  |
| CVVHD | |  | | |  | — |  |
| CVVHDF | |  | | |  | 0.83 (0.17 to 4.13) | 0.81 |
| Sex | |  | | |  |  |  |
| F | |  | | |  | — |  |
| M | |  | | |  | 1.29 (0.30 to 5.52) | 0.73 |
|  |  | |  |  |  |  |  |

The table illustrates the association between discontinuation and continuation of CKRT after reaching a T/iCa ratio ≥ 2.5 and ICU mortality, analyzed in a univariable model and after multivariable adjustment. To reduce the complexity of the model in this smaller subgroup cohort, a reduced set of covariables with a high likelihood of influencing the endpoint, as determined by a directed acyclic graph, was included. OR = Odds ratio, 95% CI = 95 % confidence interval, VIS = Vasoactive Inotropic Score, INR = International normalized ratio, CVVHD = Continuous venovenous hemodialysis, CVVHDF = Continuous venovenous hemofiltration.

| **Table S11 b. - Cleared vs. non-cleared citrate accumulation and association with mortality** | | | | | | | |
| --- | --- | --- | --- | --- | --- | --- | --- |
| **Characteristics** | | **Univariable** | | | | **Multivariable** | |
|  | | **OR**  **(95% CI)***^1^* | | | **p-value** | **OR**  **(95% CI)***^1^* | **p-value** |
| Citrate cleared | |  | | |  |  |  |
| No | | — | | |  | — |  |
| Yes | | 0.22 (0.07 to 0.60) | | | **0.005** | 0.50 (0.07 to 2.96) | 0.45 |
| Factor V, sqrt(%) | |  | | |  | 0.95 (0.45 to 1.99) | 0.89 |
| Bilirubin, log(µmol/L) | |  | | |  | 1.17 (0.55 to 2.51) | 0.67 |
| INR, log | |  | | |  | 0.02 (0.00 to 1.83) | 0.12 |
| Platelets, sqrt(G/L) | |  | | |  | 0.85 (0.65 to 1.08) | 0.19 |
| Lactate, log(mmol/L) | |  | | |  | 4.37 (1.24 to 20.5) | **0.034** |
| VIS, sqrt | |  | | |  | 0.98 (0.69 to 1.40) | 0.92 |
| Age, years | |  | | |  | 1.00 (0.94 to 1.06) | 0.93 |
| Modus | |  | | |  |  |  |
| CVVHD | |  | | |  | — |  |
| CVVHDF | |  | | |  | 2.13 (0.32 to 17.2) | 0.45 |
| Sex | |  | | |  |  |  |
| F | |  | | |  | — |  |
| M | |  | | |  | 2.46 (0.42 to 16.5) | 0.32 |
|  |  | |  |  |  |  |  |

The table presents the association between successful citrate clearance and mortality compared to those who did not achieve clearance among a subset of patients with continued CKRT after reaching a T/iCa ≥ 2.5. OR = Odds ratio, 95% CI = 95 % confidence interval, VIS = Vasoactive Inotropic Score, INR = International normalized ratio, CVVHD = Continuous venovenous hemodialysis, CVVHDF = Continuous venovenous hemofiltration.

**
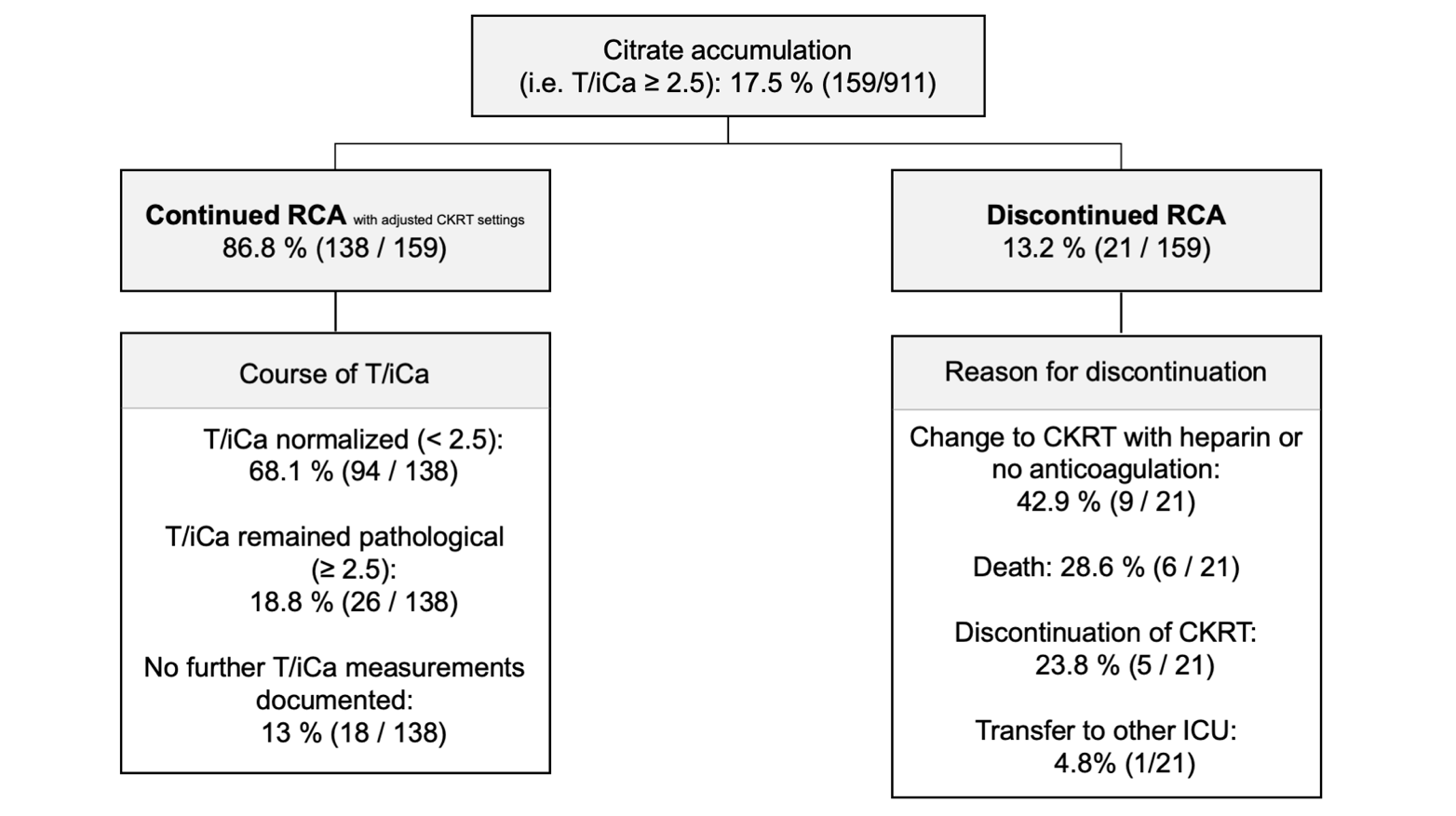
**

**Fig. S12 – Citrate accumulation and continuation of RCA-based CKRT**

The figure shows the proportion of RCA treatments that were continued or discontinued after reaching a total albumin-corrected to ionized calcium ratio (T/iCa) ≥ 2.5, the proportion of cases in which T/iCa normalised or remained pathological, and the reasons for discontinuation of RCA.
